# Prevalence, severity and early outcomes of hypoxic ischemic encephalopathy among newborns at a tertiary hospital, in northern Tanzania

**DOI:** 10.1186/s12887-017-0876-y

**Published:** 2017-05-25

**Authors:** Irene N. Simiyu, Deborah N. Mchaile, Kahindo Katsongeri, Rune N. Philemon, Sia E. Msuya

**Affiliations:** 1 Kilimanjaro Christian Medical University College (KCMUCo), Moshi, Tanzania; 2 Department of Paediatrics, Kilimanjaro Christian Medical Centre (KCMC), Moshi, Tanzania; 3 Department of Obstetrics and Gynaecology, Kilimanjaro Christian Medical Centre, Moshi, Tanzania; 4 Department of Community Health, KCMUCo, Institute of Public Health, Moshi, Tanzania; 5 Community Health Department, Kilimanjaro Christian Medical Centre, Moshi, Tanzania; 6 Department of Epidemiology & Biostatistics, KCMUCo, Institute of Public Health, Moshi, Tanzania; 7 KCMC Hospital, Moshi, Tanzania

**Keywords:** Hypoxic ischemic encephalopathy, HIE, Birth asphyxia, Neuro-muscular signs, Neonates, Early outcomes, Tanzania

## Abstract

**Background:**

Hypoxic Ischemic Encephalopathy (HIE) remains a problem of great concern worldwide especially in developing countries. The occurrence of a neurological syndrome can be an indicator of insult to the brain. We aimed to determine the prevalence, HIE proportions, neurological signs and early outcomes of newborns that developed birth asphyxia at KCMC Tanzania.

**Methods:**

A prospective study was conducted at KCMC from November 2014 to April 2015 among newborns with birth asphyxia. Sarnat and Sarnat score was used to assess newborns immediately after birth to classify HIE and were later followed daily for 7 days or until discharge.

**Results:**

Of the 1752 deliveries during the study period, 11.5% (*n* = 201) had birth asphyxia. Of the 201 newborns, 187 had HIE. Of these 187 with HIE; 39.0% had moderate HIE and 10.2% had severe HIE according to the Sarnat and Sarnat classification.

Neurological signs that were observed during the study period were; weak/absent reflexes (46.0%), hypotonia (43.3%) and lethargy (42.2%).

Mortality was 9.1% among the 187 newborns with HIE. Mortality was higher among newborns with severe HIE 84.2% (16/19) compared to those with moderate HIE 1.4% (1/73). On the 7th day after delivery, 17.1% (32/187) of the newborns did not show any change from the initial score at delivery.

**Conclusion:**

Prevalence of birth asphyxia is high in our setting and most of the newborns (49%) end up with moderate/severe HIE. Good obstetric care and immediate resuscitation of newborns are vital in reducing the occurrence of HIE and improving the general outcome of newborns.

## Background

Birth anoxia remains a vital cause of morbidity and mortality in neonates. Hypoxic ischemic encephalopathy affects the tissues of the body and can lead to permanent brain damage. Hypoxic ischemic encephalopathy results from lack of oxygen before, during or after birth [1].

The countdown report of 2015 estimated that neonatal deaths account for 45% of the 5.9 million child deaths that occurred in 2015 globally. Birth asphyxia is responsible for a large number of neonatal deaths after preterm births [2]. Perinatal asphyxia is thus a serious problem for child survival globally, more in developing countries. Apart from increased mortality, perinatal asphyxia results in serious neurological consequences ranging from cerebral palsy and mental retardation to epilepsy [3]. Reaching the sustainable development goal of 2030 of reducing preventable neonatal mortality to less than 12 deaths per 1000 live births requires research and evidence interventions that target neonatal period [4].

Deaths due to perinatal asphyxia causes shock and pain to the mother and family at large. However, it can sometimes be avoided by close monitoring during the birth process with several assessments which include umbilical pH, 1 h post delivery blood gas, Apgar scores and neurological changes ranging from twitching, hypotonia, and seizures [3]. An Apgar scores at 5 min provides useful prognostic data before other evaluations are available. Low Apgar scores at 1, 5 and 10 min have been found to be markers with possible increased risk of death or chronic motor disability [3].

Neonatal encephalopathy preceded by perinatal hypoxic ischemic insult is a main contributor to global child mortality and morbidity. Brain injury in infants is a process that evolves over hours to days providing an opportunity for neuro-protective interventions. Advances in neuroimaging, techniques in monitoring of the brain and tissue biomarkers have improved the ability to diagnose, monitor and care for newborn infants. However, challenges remain in the availability of these imaging modalities in low income settings like Tanzania [5].

The gap between developed and developing countries with regards to neonatal mortality still remains wide. A child born in a developing country is 14 times more likely to die during the first 28 days of life than a baby born in a developed country; with sub-Saharan Africa and South East Asia being most affected [6]. These could be attributed to the lack of investigating and monitoring modalities. The aim of this study was to determine the prevalence, describe the severity and early neonatal outcomes of hypoxic ischemic encephalopathy among newborns with birth asphyxia (Apgar score < 7 at 5 min) at KCMC referral hospital born between November 2014 and April 2015.

## Methods

### Study design and area

This was a prospective study which enrolled newborns immediately after delivery and followed them daily for a period of 7 days after delivery. The study was conducted at the labor ward and newborns followed up at the neonatal ward (P3) at KCMC referral hospital situated in northern Tanzania. KCMC is a teaching and referral hospital in Moshi urban district. As a zonal referral center, KCMC receives normal and complicated cases from the local community in Kilimanjaro region and nearby regions in the Northern Tanzania and from nearby districts in Kenya.

The neonatal ward at KCMC hospital is a modest 6 room ward. Of these, 3 rooms are for acute cases with approximately 60 cots whilst the other 3 rooms have 15beds mainly for recovering newborns awaiting discharge. The neonatal ward admits a considerable number of neonates per year with most of those delivered being from within the facility. The unit does not have high tech advanced diagnostic facilities like most of SSA countries. Babies with asphyxia as assessed by the Sarnat and Sarnat staging are usually kept in the cool area of the ward where heaters are not switched on. Management offered is usually observation, oxygen therapy depending on the severity of hypoxia and prevention of sepsis. Blood PH, mechanical ventilation, Electroencephalogram (EEG), brain imaging or therapeutic cooling/hypothermia is not available at this set up.

### Study population, sampling and enrollment procedure

The study population was all newborns seen at KCMC between November 2014 and April 2015 who had an Apgar score < 7 at the 5th minute. Newborns with birth asphyxia but with obvious congenital malformations were excluded from the study.

The formula for precision by Leslie Kish was used to calculate the sample size [7]. The minimum sample size required was 327 based on prevalence of HIE of 30.9% observed in Dar es Salaam [8], confidence interval of 95% and error set at 5%.

At the labor ward a standardized data extraction sheet was used to collect information from the patients’ files and records of birth registry. Information collected was on maternal demographic information, gestation age at delivery based on last menstrual period, duration of labor, mode of delivery, birth complications, birth weight, Apgar score and occurrence of signs of HIE. Sarnat and Sarnat classification (Table 1), was used to record neurological signs and classify the severity of HIE of the newborns with birth asphyxia immediately after birth, and daily up to 7th day after birth or discharge from the neonatal ward.

**Table 1:** Sarnat and Sarnat Scoring System

	1	2	3	4	5	6	7	1	2	3	4	5	6	7	1	2	3	4	5	6	7
	1							2							3						
Consciousness	Hyper alert							Lethargic							Stuporous						
Neuromuscular	Normal							Mild hypotonia							Flaccid						
Reflexes	Weak > Normal							Weak							Absent						
Pupils	Dilated							Constricted							Variable						
Heart rate	Tachycardia							Bradycardia							Variable						
Secretions	Sparge							Profuse							Variable						
GIT motility	Normal							Increased							Variable						
Convulsions	None							Common							Common early less common later						
Duration	24 h							2-14 days							Weeks						

### Data processing and analysis

Data collected was entered and analyzed using the statistical package for the social science (SPSS) version 20. Categorical data was summarized using proportions, while mean and median with their measures of dispersion were used to summarize continuous data.

### Categorization of severity of HIE

Newborns who were born with birth asphyxia were classified according to the Sarnat and Sarnat scoring criteria for HIE. The tool has 8 items which are assessed. Neonates scoring 1-10 were classified as having mild HIE, 11-14 as moderate HIE while those scoring 15-22 were classified as having severe HIE. The highest score obtained on any of the 7 days of follow up was used to assess the severity of birth asphyxia.

### Ethical approval

Approval to conduct the study was obtained from KCMUCo Research and Ethics Committee. Permission to conduct the study was also obtained from the Executive Director of KCMC Hospital and the heads of department of Pediatrics and Obstetrics and Gynecology. Consent was obtained from mothers. All newborns were managed in the neonatal ward following the standardized protocol for the unit.

## Results

### Prevalence of birth asphyxia

A total of 1752 newborns were delivered at the labor ward during the study period (November 2014 – April 2015). Of these newborns, 201 (11.5%) had birth asphyxia which was defined by an APGAR score less than 7 at 5 min after birth. Of the 201 newborns with asphyxia, 187 had hypoxic ischemic encephalopathy (HIE) giving a prevalence of 10.7% (187/1752). These 187 neonates with HIE were followed up daily for 7 days or until discharge if it happened before 7 days.

### Characteristics of the participants

The median gestation age at delivery of the 187 newborns was 38 weeks (range 27 - 43). Their median birth weight was 2900 g (range 1100 - 5000). More than half 52.9% of the participants were females and delivered by cesarean section (70.1%), see Table 2. Of 131 cesarean sections, 96% were emergencies.

**Table 2:** Characteristics of newborns with hypoxic ischemic encephalopathy at KCMC between Nov 2014 and April 2015 (*N* = 187)

Variable	Frequency	%
Sex		
Male	88	47.1
Female	99	52.9
Birth Weight		
Low Birth weight (<2500 g)	63	33.7
Normal (2500-4500 g)	123	65.8
Over weight (>4500 g)	1	0.5
Gestational age at delivery		
Pre term (<37 weeks)	61	32.6
Term (37-42 weeks)	121	64.7
Post Term (>42 weeks)	5	2.7
Apgar score at 5 min		
4˗6	176	94.1
1˗3	11	5.9
Referral		
Yes	123	65.8
No	64	34.2
Mode of delivery		
Spontaneous vaginal delivery	52	27.8
Cesarean section	131	70.1
Instrumental delivery	4	2.1

### Severity of HIE

According to the Sarnat and Sarnat classification, majority of the 187 newborns with HIE had mild HIE 50.8% (*n* = 95) and 10.2% (19) had severe HIE, Table 3.

**Table 3:** Proportions of newborns with asphyxia who had mild, moderate or severe HIE per Sarnat and Sarnat classification (*N* = 187)

Variable	Frequency	%
Mild (1-10)	95	50.8
Moderate (11-14)	73	39.0
Severe (15-22)	19	10.2

Higher proportion of severe HIE was noted among females 11.1% (*n* = 11) compared to males 9.1% (*n* = 8), but this difference was not a significant difference OR 1.25 (95% CI: 0.48 – 3.26). Severe HIE was also observed among LBW (< 2500 g) 14.3% than normal weight (≥ 2500 g), OR 1.90 (95% CI: 0.73 – 4.95).

### Common neurological signs observed

**Fig. 1:**
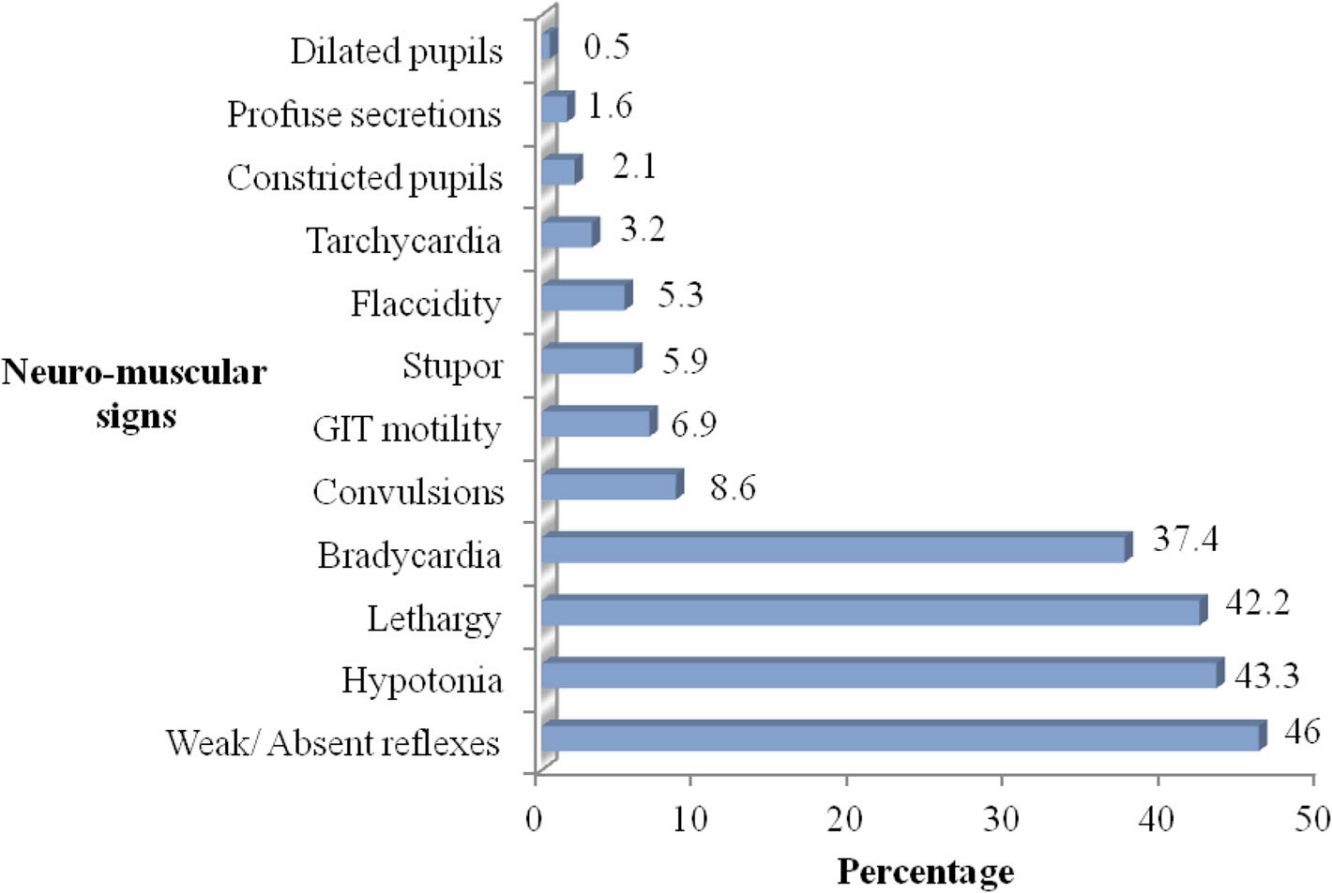
Neurological signs seen among the newborns who had HIE

Figure 1 shows common clinical signs that were observed in the newborns with HIE. Weak/absent reflexes (46.0%), hypotonia (43.3%) and lethargy (42.2%) were the most common observed signs. Among the least occurring signs observed were; dilated pupils (0.5%), profuse secretions (1.6%), and constricted pupils (2.1%).

### Early outcomes of newborns with HIE

Majority 73.7% (*N* = 138), of the 187 neonates with HIE were discharged within the 7 days of observation and 17.1% (*N* = 32) showed no clinical response. A total of 17 newborns died during the time of study which accounted for a general mortality of 9.1% among the neonates with HIE. Majority of the newborns who died were those with severe HIE 84.2%, than those with moderate HIE 1.4%, with no deaths among neonates with mild HIE, Table 4. Newborns with low birth weight had significantly higher mortality (15.9%) compared to those born with a normal weight (5.7%) [OR 3.15, (95% CI: 1.1 -8.7)].

**Table 4:** Immediate outcomes by HIE classification, level of maturity and birth weight among newborns with birth asphyxia at KCMC hospital

	*N*	Discharge(*n* = 138)	Death (*n* = 17)	No Response in 7 days (*n* = 32)
Mild HIE	95	94 (98.9%)	0 (0.0%)	1 (1.1%)
Moderate HIE	73	44 (60.2%)	1 (1.4%)	28 (38.4%)
Severe HIE	19	0 (0.0%)	16 (84.2%)	3 (15.8%)
Premature (<37 weeks)	61	26 (42.6%)	7 (11.5%)	28 (45.9%)
Full term (37+)	126	112 (88.8%)	10 (7.9%)	4 (3.2%)
Low birth weight (<2500 g)	63	27 (42.9%)	10 (15.9%)	26 (41.3%)
Normal (2500-4500 g)	124	110 (89.4%)	7 (5.7%)	6 (4.9%)

Marked improvement and discharge was observed in newborns that had mild and moderate HIE (98.8 and 60.2%) respectively.

Neonates with moderate HIE and premature newborns were still hospitalized by the 7th day with no response or change in HIE status compared to others, Table 4.

## Discussion

In this study 11.5% of the neonates had birth asphyxia and 10.7% were found to have HIE. The prevalence of HIE observed is lower compared with the findings of a study done in Tanzania at Muhimbili National Hospital in 2007 where the prevalence was found to be 30.9% [8]. It’s however higher than among the studies done in high income countries. In Spain the prevalence of HIE was found to be 2.42 per 1000 infants between 2000 and 2008 [9]. Birth asphyxia is among the leading causes of death in the neonatal period and in Tanzania it contributes to 23% of the neonatal deaths. Apart from mortality, neonates who develops HIE have a risk of serious long term neuro-motor sequelae like cerebral palsy and epilepsy among the survivors. Hence improvement in monitoring of mothers in labor and of the newborns with HIE should be strengthened in our setting.

Most newborns who had HIE in this study had the mild form (50.8%). This is similar to findings done in South India where the proportion of newborns with mild HIE was 56% [10] and from another study conducted in Rotunda hospital in Ireland where of the 237 newborn assessed, 65.4% had the mild form of HIE [11]. Of note to practitioners’ majority (98%) of newborns with mild HIE in this study improved and were discharged similar to observations at Muhimbili National Hospital in Tanzania, in Pakistan and India respectively where a total of 92.3% of the newborns were discharged to their mothers [5, 10, 12]. This indicates that with the appropriate care newborns with HIE can improve sufficiently to be discharged early.

During the study period, the most commonly occurring neurological signs were; weak/absent reflexes, hypotonia and lethargy. This compares closely with the findings from Liaquat University Hospital in Pakistan where among the most observed signs were depressed neonatal reflexes, lethargy and papillary abnormalities [12]. However, in Besat hospital in Iran, seizures (9.1%) were a common presenting sign, unlike in this study where only 8.6% of the participants had seizures being among the least occurring sign [13]. Presence of these signs should alert the health care providers at all levels to monitor newborns closely or timely referral from lower level health facilities.

Overall mortality observed among newborns with HIE was 9.1%. This finding is within observations of previous studies at Mulago Hospital in Uganda 12.9% [14], in Cameroon 10% [6], at tertiary hospital in Johannesburg South Africa 14.3% [3], at Liaquat teaching hospital in Pakistan 15% [12] and at Ayub Teaching hospital in Pakistan 16% [1]. Mortality was high among LBW newborns and those with severe HIE. This means strategies to reduce low birth weight should be reinforced. Further improving quality of monitoring labor cannot be overemphasized to prevent intrapartum related asphyxia.

Nearly 4 in 10 newborns with mild HIE had not responded by 7th day in the ward, so as 41% of LBW and 46% of preterm newborns. At seventh day of follow up, overall 17.1% (32) of the newborns had shown no response compared to initial assessment. The risk of long term sequelae is high in these 3 groups and this shows the need for long term follow up of children with HIE.

### Limitations

Inclusion of preterm babies could have influenced some of the findings especially the neurological signs and mortality. Clinical assessment alone was used to categorize the severity of HIE which does not give a complete picture of the effect of hypoxia on the brain. Future studies should complement clinical assessment with EEG or CT scan for complete monitoring and evaluation of newborns with HIE.

## Conclusions

The overall prevalence of hypoxic ischemic encephalopathy at KCMC tertiary hospital was found to be 10.7%. Most newborns presented with the mild form of HIE (50.8%) and were discharged to their mothers during the study period. Mortality during the first week of life was 9.1%, highest among neonates with the severe form of HIE and with low birth weight. At 7th day approximately 4 out of 10 neonates with moderate HIE, who were preterm or LBW did not show any improvement in response to treatment offered.

We recommend widespread usage and monitoring of newborns with asphyxia using the Sarnat and Sarnat classification chart in low income settings where sophisticated monitoring is not possible. Essential practices to improve monitoring of labor (before and during) are needed. As well as much emphasis on appropriate care of newborns with HIE.

### Limitations

Usage of the Sarnat and Sarnat score as compared to other scored that have been clinically validated may have lead to either overestimation or under-estimation of the prevalence.

## Abbreviations

EEG: Electroencephalogram
HIE: Hypoxic Ischaemic Encephalopathy
KCMC: Kilimanjaro Christian Medical College
KCMUCo: Kilimanjaro Christian Medical University College
LBW: Low birth weight
